# Case Report: *Streptococcus alactolyticus* as a Rare Pathogen of Mitral Endocarditis

**DOI:** 10.3389/fcvm.2021.648213

**Published:** 2021-04-29

**Authors:** Mattia Vinciguerra, Valeria Santamaria, Silvia Romiti, Mizar D'Abramo, Gianmarco Toto, Antonio De Bellis, Gloria Taliani, Giuseppe Sangiorgi, Ernesto Greco

**Affiliations:** ^1^Department of Clinical, Internal Medicine, Anesthesiology and Cardiovascular Sciences, Sapienza University of Rome, Rome, Italy; ^2^Department of Cardiology and Cardiac Surgery, Casa di Cura “S. Michele”, Maddaloni, Italy; ^3^Hepatology Unit, Department of Translational and Precision Medicine, Sapienza University of Rome, Rome, Italy; ^4^Division of Cardiology, Department of Biomedicine and Prevention, University of Tor Vergata, Rome, Italy

**Keywords:** infective endocarditis, *Streptococcus alactolyticus*, mitral valve, mitral valve regurgitation, *Streptococcus bovis*

## Abstract

*Streptococcus bovis/Streptococcus equinus* complex (SBSEC) is a group of *non-enterococcal group D Streptococci* that colonizes both humans and animals. Due to gastrointestinal disease, they can switch in opportunistic pathogens passing through intestinal mucosal barrier and may cause bacteremia and distant organs damage. Despite infective endocarditis (IE), extra-cardiac manifestations of organs damage include osteoarticular infections, meningitis, and biliary infections among others; moreover, the association with colonic pathological lesions has been largely described. *Streptococcus alactolyticus* as a species included in SBSEC may share pathophysiological similarities, although it represents an extremely rare cause of distant organ infections, being reported in literature as causative agent of IE in only two other cases. We describe a case of 69-year-old male admitted to our institution due to mild–moderate dyspnea and fever, affected by cervico-brachialgia for 3 weeks. *Streptococcus alactolyticus* was identified as causative agent of IE on the mitral valve, causing severe regurgitation.

## Introduction

Every year, between two and six people per every hundred-thousand inhabitants worldwide suffer from infective endocarditis (IE) (1). When IE is not properly treated, significant complications arise. Methods for early diagnosis have been developed, opening the opportunity for better surgery timings (which would have a very relevant impact on patients' evolution). However, the 1-year mortality average rate has not improved over the last two decades (2).

The rheumatic chronic disease remains the main cause in low-income countries. Despite the large use of antibiotics in industrialized countries, *Streptococci*, derived from oral microbiome, it still represents the main cause. The IE that is caused by *Staphylococcus Aureus* and coagulase-negative *Staphylococci* is found significantly on patients with intravenous drug use history, prosthetic-valve or device implanted, or somehow affected by other comorbidities. *Streptococcus bovis* (group *D Streptococci*) is more frequently found on elderly patients (1).

*Streptococcus alactolyticus* belongs to the *S. bovis* complex group and can be a cause of IE, although this is considered extremely rare (3).

Our case report will address an IE case on a 69-year-old male patient, caused by *Streptococcus alactolyticus*, a very unusual pathogen, who presented spondylodiscitis as the early manifestation of the infectious process.

## Methods

### Ethics

Written informed consent was obtained from the individual for the publication of any potentially identifiable images or data included in this article.

## Case Report

A 69 years-old fit male with an active life was admitted to our institution. He suffered from cervico-brachialgia, arisen 3 weeks before, and fever. He affirmed lack of appetite and weight losses during the last month, together with mild–moderate dyspnea on the previous 4 days.

His clinical history briefed hepatic steatosis and surgery 30 years before due to left colon cancer.

**Figure 1:**
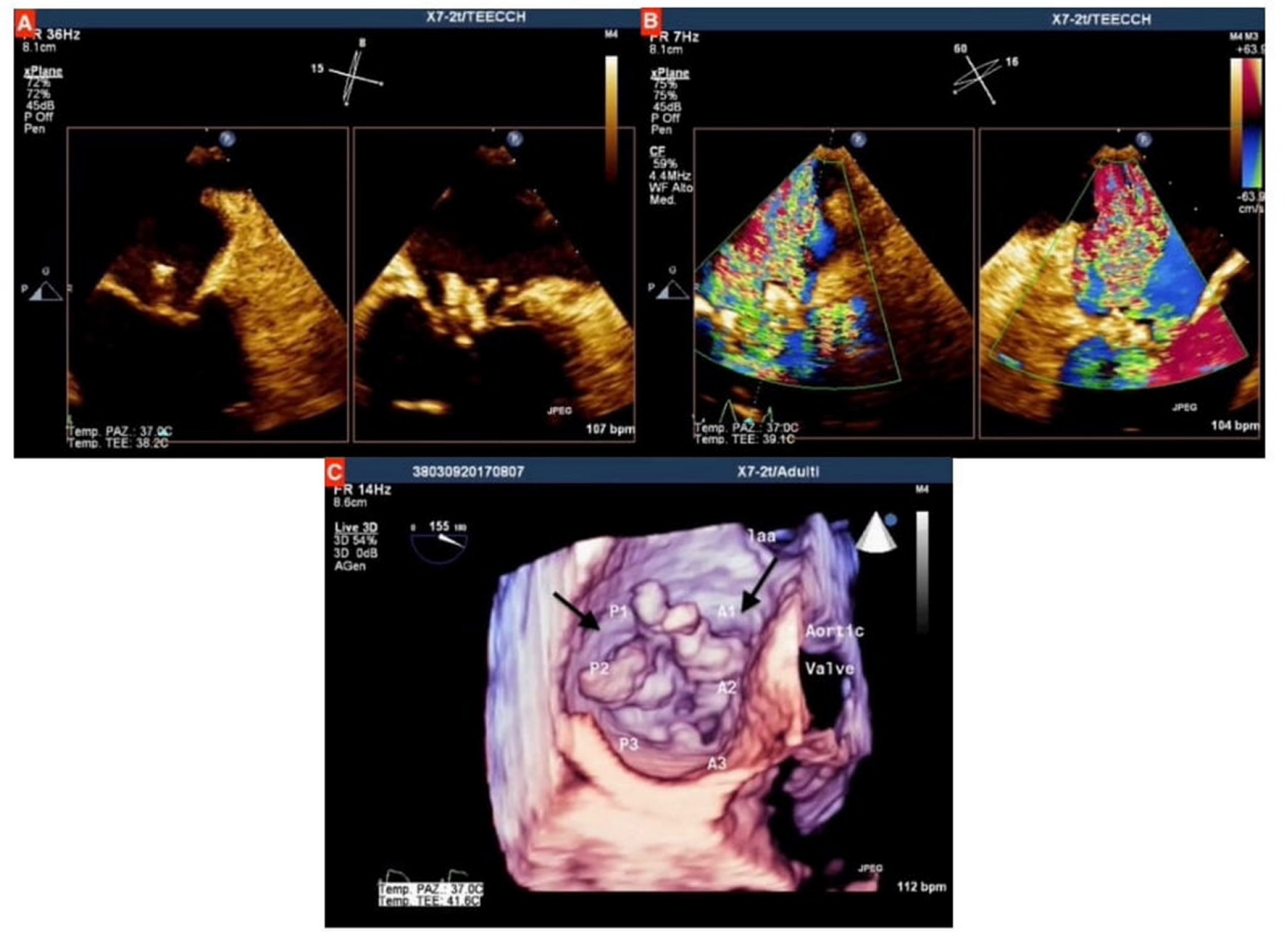
**(A)** Qualitative assessment of mitral valve at transesophageal echocardiogram (TEE), showing the two vegetations on both anterior and posterior mitral valve leaflets, which protrude toward left atrium during systole; **(B)** Assessment with color doppler showing severe mitral regurgitation; **(C)** three-dimensional assessment at TEE, the black arrows indicate the two vegetations on mitral leaflets.

The transthoracic echocardiogram (TTE) diagnosed a severe mitral regurgitation (MR) due to IE; it was confirmed by transesophageal echocardiogram (TEE), which showed two vegetations on the atrial face of mitral valve, sized 11 × 10 mm on the posterior leaflet and 7 × 5 mm on the anterior leaflet (Figure 1). The blood culture was positive for *Streptococcus alactolyticus*, and consequently, the following intravenous antibiotic therapy was prescribed: Gentamicin (80 mg × 3 every day for 2 weeks) and Ceftriaxone (2 g × 2 every day for 4 weeks).

The cervical magnetic resonance imaging (MRI) confirmed the presence of non-specific inflammation of vertebral soma C5 and C6, endorsing spondylodiscitis.

The total body computed tomography (CT) was remarkable for a hypodense area of 13 mm in the spleen and a lumbar lymph node of extended size. This was probably related to infarction derived from a septic embolism.

To assess the possible sites of infection, the patient underwent odontoiatric evaluation. The result was negative for dental foci, but the musculoskeletal and joint ultrasound of his right ankle and foot showed a synovitis of phalangeal metatarsal joint.

A colonoscopy did not show any pathological lesions beside the left hemicolectomy. The second TTE showed a size reduction of the valve vegetations. Nevertheless, owing to the presence of severe MR and the high risk of embolism, cardiac surgery was scheduled.

**Figure 2:**
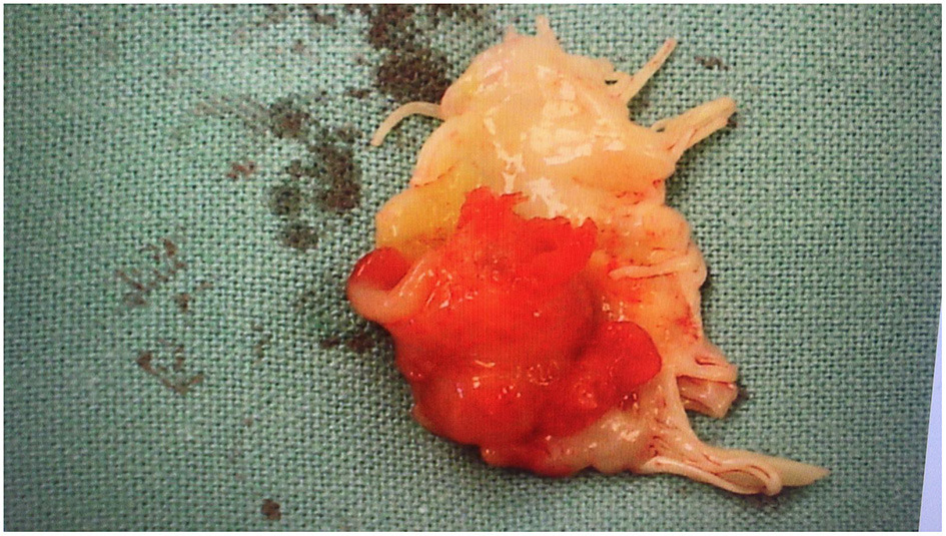
Large vegetation on the atrial face of anterior mitral valve leaflet.

The patient underwent surgery 16 days after the beginning of the antibiotic therapy, started and later confirmed with blood cultures results, the day after the admission at the Hospital. Intra-operatively, the diagnosis of IE was confirmed, identifying huge vegetations on the atrial face of mitral valve leaflets (Figure 2).

The mitral valve was replaced for a tissue valve (Perimount Magna 31, Edwards Life Sciences Irvine CA). Histology did not reveal any polymorphs, no organisms were identified on Gram stain, and routine cultures failed to grow any organism because of the antibiotic therapy. *Streptococcus alactolyticus* was identified on mitral valve tissue using polymerase chain reaction (PCR).

The post-operative course was complicated by a Third-Degree Atrioventricular Block requiring a DDD pacemaker (PMK) implant, after a watchful and wait period in which temporary epicardial pacing was used. Standard antibiotic prophylaxis with cefazolin was used before PMK implantation in addition to tailored therapy for IE. A pericardial effusion required sub-xiphoid surgical drainage.

The intravenous antibiotic therapy was continued after the operation for 12 days and stopped 5 days before discharge.

The patient was discharged with a good clinical condition on his 17th post-operative day.

## Discussion

The *Streptococcus bovis/Streptococcus equinus* complex **(**SBSEC**)** is a group of *non-enterococcal group D Streptococci* that colonizes the gastrointestinal tract as commensal in humans and animals and is involved in food fermentation (4). The incidence of colonization in humans increases in rural areas, since it may be associated to contact with animal feces and fermented food products (5).

SBSEC comprises different species that can be divided into two biotypes, according to their capacity to ferment mannitol (4, 6).

In literature, *Streptococcus alactolyticus* species is classified as biotype I of *S. bovis* (7). However, the correct classification is still not clearly defined (5).

The clinical importance of SBSEC is related to the possibility to switch from a common condition of commensal bacteria to become an opportunistic pathogen due to gastrointestinal disease. The bacterial translocation from gastrointestinal tract is linked to septicemia, urinary tract infections, IE, biliary infections, meningitis, and osteo-articular infections (OAIs) (5, 8).

The SBSEC is recognized as an infective cause of endocarditis in up to 6% of the overall confirmed cases, with an increasing incidence compared to the past (9).

Besides subjects over 65 years old with healthy heart valves, patients with clinical conditions impacting on the infective occurrence, such as congenital heart defects, prosthetic valve, diabetes and cross infections, are commonly involved (9).

The perfect substrate for the establishment of IE is represented by a damaged endothelial tissue of valve (3). The injured tissue is a focus for the recruitment of platelets and fibrin increasing the capability of bacterial adherence. The pathogens, during bacteremia, colonize the valvular tissue and once established, they form a biofilm that works as a shield against immune attacks (5).

In this context, TTE is an essential diagnostic tool allowing a detailed and non-invasive first approach to cardiac valvular disease and in particular allowing surgery plan decision in mitral valve surgery (10).

In literature, the *Streptococcus alactolyticus* is documented as a causative agent of IE, identified by blood cultures, only in other three cases, representing an extremely rare occurrence.

Almeida et al. has described a 65-year-old female affected by IE on both aortic and mitral valve complicated by septic embolism and left middle cerebral artery aneurysm. The patient developed neurological symptoms after a dental procedure, which acted as causative agent first for bacteremia and then for IE (11). The rate of embolic events in SBSEC endocarditis ranges between 9 and 55% (12).

Cekmen et al. (13) has described a case of IE by *Streptococcus alactolyticus* involving also the aortic and mitral valves but without evidence of septic embolism. The patient had undergone, a month earlier, a coronary artery bypass graft (CABG) operation.

Recently, Mylonas et al. (14) reported a similar case of IE caused by *Streptococcus alactolyticus* affecting a prolapsing mitral valve; they further reviewed literature describing cases aforementioned.

In these three cases, as well as in our reported one, the treatment was initially based on the antimicrobial sensitivity of *Streptococcus alactolyticus*, setting an appropriate therapy with Ceftriaxone. Then, the patients underwent surgery due to severe valvular regurgitation. Additionally, the vegetations were described by TEE as of significant dimension (more than 1 cm), underlying the molecular mechanism based on the presence of pili phase variation and the expression of a capsular polysaccharide (5).

The same mechanism is frequently involved in several extra-cardiac conditions caused by SBSEC (3). *S. alactolyticus* has been described as a precipitator agent in a case of severe diabetic ketoacidosis and in two cases of extra-cardiac infection: a fulminant neonatal sepsis and a recent case of neonatal meningitis (15, 16).

The septic arthritis and osteomyelitis are frequently described as an initial manifestation of IE caused by SBSEC, and in particular by the species *Streptococcus gallolyticus*, subspecies gallolitycus (SGG) (17). In our case, the patient presented cervical spondylodiscitis from 3 weeks prior to cardiac involvement.

The OAI may represent a first indicator of a systemic infective process such as a colorectal cancer (18). In fact, a strong association between SBSEC infection and gastrointestinal disease has been known for this form of cancer (19).

The etiologic mechanism is based on the capacity of the pathogens to cause chronic inflammation and to elude the immune system (5).

The current recommendations suggest to perform a colonoscopy in patients with IE or other infections caused by the *S. bovis* group, in particular by SGG, which is associated in two-thirds of the cases with colonic cancer (5).

Our patient underwent a colonoscopy without evidence of any pathological lesion. He had a history of left colonic cancer that had arisen when he was 39 years old, not presenting remarkable risks factors. It is difficult to establish whether a genetic predisposition or a bacterial causative agent existed, but we can hypothesize that the surgical treatment with a hemicolectomy might have contributed to develop a dysbiosis of intestinal microbiota, leading to a bacterial translocation.

The typical distinct properties of SBSEC may provide a clear selective advantage in the gut, allowing when facilitated by intestinal environment conditions, such as potentially due to surgical injury, the translocation toward the target organ with collagen-rich surfaces such as heart valves (20).

## Conclusion

This case emphasizes the importance of a correct diagnosis to optimize the treatment of IE. Endocarditis caused by SBSEC members (in particular the ones belonging to biotype I) has to be considered as a peculiar systemic infective disease with others target organs rich in the collage surface. Appropriate techniques of imaging are mandatory and help to correctly assess the progression of the disease: from the initial pathological lesion in the gut, through the endocardial or disc involvement until the peripheral septic emboli.

## Data Availability Statement

The original contributions presented in the study are included in the article/supplementary material, further inquiries can be directed to the corresponding author/s.

## Author Contributions

MV and VS collecting data about patient clinical informations. SR, MD'A, and GTo contributed in writing and revising manuscript. AD, GTa, GS, and EG critically revised the manuscript. All authors contributed to the article and approved the submitted version.

## Conflict of Interest

The authors declare that the research was conducted in the absence of any commercial or financial relationships that could be construed as a potential conflict of interest.
